# Lung nodule localization and size estimation on chest tomosynthesis

**DOI:** 10.1117/1.JMI.12.S1.S13007

**Published:** 2024-10-28

**Authors:** Micael Oliveira Diniz, Mohammad Khalil, Erika Fagman, Jenny Vikgren, Faiz Haj, Angelica Svalkvist, Magnus Båth, Åse Allansdotter Johnsson

**Affiliations:** a University of Gothenburg, Sahlgrenska Academy, Institute of Clinical Sciences, Department of Radiology, Gothenburg, Sweden; b Region Västra Götaland, Sahlgrenska University Hospital, Department of Radiology, Gothenburg, Sweden; c University of Gothenburg, Sahlgrenska Academy, Institute of Clinical Sciences, Department of Medical Radiation Sciences, Gothenburg, Sweden; d Region Västra Götaland, Sahlgrenska University Hospital, Department of Medical Physics and Biomedical Engineering, Gothenburg, Sweden

**Keywords:** digital chest tomosynthesis, lung nodule measurements, area, mean diameter

## Abstract

**Purpose:**

We aim to investigate the localization, visibility, and measurement of lung nodules in digital chest tomosynthesis (DTS).

**Approach:**

Computed tomography (CT), maximum intensity projections (CT-MIP) (transaxial versus coronal orientation), and computer-aided detection (CAD) were used as location reference, and inter- and intra-observer agreement regarding lung nodule size was assessed. Five radiologists analyzed DTS and CT images from 24 participants with lung $\text{nodules}\geq 100\;{\mathrm{mm}}^{3}$, focusing on lung nodule localization, visibility, and measurement on DTS. Visual grading was used to compare if coronal or transaxial CT-MIP better facilitated the localization of lung nodules in DTS.

**Results:**

The majority of the lung nodules (79%) were rated as visible in DTS, although less clearly in comparison with CT. Coronal CT-MIP was the preferred orientation in the task of locating nodules on DTS. On DTS, area-based lung nodule size estimates resulted in significantly less measurement variability when compared with nodule size estimated based on mean diameter (mD) (p<0.05). Also, on DTS, area-based lung nodule size estimates were more accurate ($\mathrm{SEE}=38.7\;{\mathrm{mm}}^{3}$) than lung nodule size estimates based on mean diameter ($\mathrm{SEE}=42.7\;{\mathrm{mm}}^{3}$).

**Conclusions:**

Coronal CT-MIP images are superior to transaxial CT-MIP images in facilitating lung nodule localization in DTS. Most $\text{nodules}\geq 100\;{\mathrm{mm}}^{3}$ found on CT can be visualized, correctly localized, and measured in DTS, and area-based measurement may be the key to more precise and less variable nodule measurements on DTS.

## 1 Introduction

Digital tomosynthesis (DTS) of the chest is a relatively modern modality, based on conventional X-ray techniques, which allows superior visualization of anatomical structures and pathological findings in multiple sections that would otherwise be seen in the same projection in a standard posterior-anterior chest X-ray.^1^^,^^2^ The ability to isolate small structures is ideal for the visualization of lung nodules. A lung nodule is a relatively well-defined approximately rounded opacity measuring up to 3 cm in diameter within the lung parenchyma.^3^ It is known that nodule size, morphology, nodule location, and nodule growth rate are characteristics associated with the risk of malignancy.^4^ Consequently, nodule size estimate is an important factor in nodule assessment in all major recommended algorithms.^5^^–^^7^

Lung nodules can be divided into three size categories: small, intermediate, and large. In general, small nodules ($<100\;{\mathrm{mm}}^{3}$) can be discharged or followed with longer intervals, intermediate nodules (100 to $250\;{\mathrm{mm}}^{3}$) need regular follow-up, and larger nodules ($\geq 300\;{\mathrm{mm}}^{3}$) need further assessment.^4^^,^^5^ The recommended tool for follow-up is computed tomography (CT).^5^^,^^6^ However, one problem with follow-up CT examinations is that the radiation associated with CT per se is a risk factor for cancer.^8^

DTS has been proposed as an alternative to CT for lung nodule follow-up,^9^ and the technique has been reported to have the potential to become a first-line screening tool for lung cancer.^2^ DTS allows visualization of the lungs in section images with less radiation compared with CT.^10^ However, there are some limitations associated with DTS, for instance, limited depth resolution and high sensitivity for artifacts from medical devices. The technique comes with a lower cost when compared with CT, and this could be of importance in centers with limited accessibility to CT scans.^11^

Lung nodules can be measured with one-dimensional measurements such as diameter, two-dimensional measurements such as area, and three-dimensional measurements such as volume.^12^ Measuring the diameter is the most common way to document lung nodule size in a radiological report. The recommendation from the Fleischner Society is to report the mean diameter (mD) based on the longest and its perpendicular diameter of the nodule independently of the plane (axial, coronal, or sagittal).^5^^,^^6^^,^^13^ However, diameter measurements might be unreliable for the assessment of small nodules both on CT^14^ and on DTS.^9^

Due to the limitations of one-dimensional measurements, some efforts have been made to incorporate area measurement as a way to provide more information about the lung nodules.^15^ However, with the introduction of multiplanar reformations (MPR) of CT images the hot topic in research soon evolved to three-dimensional measurements of lung nodule volume. Some guidelines pointed out volume as the best way to document lung nodules, not only just during the first report but also during follow-up.^16^ Two of those are the recommendations from the Fleischner Society and the British Thoracic Society, both recognizing the importance of volumetry for the measurement and management of lung nodules.^5^^,^^6^ Volumetry is the principal method for measuring solid nodules according to the British Thoracic Society recommendations and the European Lung Cancer Screening position statement.^17^ Despite these statements even 3D measurements have multiple limitations. Image acquisition, reconstruction method, CT-slice thickness, and variability between the breath-holds of the patients can influence the precision of volume measurements.^18^ A good nodule segmentation is needed to perform volumetry measurements,^16^ and the same software should be used when performing automated or semi-automated volumetry both for the initial examination and for the follow-ups.^4^^,^^19^ Volume cannot be measured on DTS, and consequently, nodule measurements must be performed using diameter or area. To the best of our knowledge, there are no previous studies about area measurement of lung nodules on DTS images of the chest.

If DTS should be considered as a follow-up examination for lung nodules it is important that radiologists are confident in localizing a nodule known from CT in a DTS examination. As far as we are aware, the confidence of radiologists in translating the correspondence of anatomical location of lung nodules on CT to the corresponding nodule on DTS has not been studied before. However, experts in the field have constructed ground truth regarding nodule localization in detection studies.^20^^–^^22^

The role of CT maximum intensity projections (CT-MIP) has been studied by others,^23^ concluding that CT-MIP images allow high diagnostic accuracy in the diagnosis of malignant pulmonary nodules. The best inter-observer agreement has been shown for axial MIP reconstruction.^24^ However, a comparison between coronal and transaxial CT-MIP regarding their usefulness in the process of localizing a nodule known from CT in a DTS examination has, to the best of our knowledge, not been investigated previously.

Consequently, the aims of the present study were to investigate (1) the preferred orientation of CT-MIP for facilitating localization of lung nodules in DTS, (2) the visibility of nodules in DTS in direct comparison to CT, and (3) which nodule size measurement technique (diameter or area) results in the smallest measurement variability in DTS.

## 2 Materials and Methods

### 2.1 Image Data

Twenty-four randomly selected cases containing lung nodules were selected from the Swedish Cardiopulmonary Bioimage Study (SCAPIS), a population-based study with participants recruited from six university cities.^25^^,^^26^ These 24 cases were composed of 14 males and 10 females, aged between 50 and 64, with a BMI variation between 22.4 and 35.3. Information about smoking habits was not available for the present study. SCAPIS was approved as a multi-center study by the regional ethical review board in Umeå (#2010/228-31M). Participants in the Gothenburg cohort underwent a DTS examination in conjunction with the chest CT performed in SCAPIS, and participants with lung nodules were invited to a study in which DTS were performed in addition to the chest CT performed clinically for lung nodule follow-up. The follow-up study was approved by the regional ethical review board in Gothenburg (#521-13). Both studies adhered to the Declaration of Helsinki, and informed consent was obtained from all participants.

All CT examinations in SCAPIS were performed with a Somatom Definition Flash (Siemens Healthcare, Forchheim, Germany). Details have been described previously.^25^ The detector configuration was 128×0.6  (mm), tube voltage 120 kV, tube current (mA) or reference dose for care dose 4D 25, rotation time 0.5 s, pitch 0.9, and matrix size 512×512. The median effective dose for the pulmonary imaging to the SCAPIS cohort was 2 mSv. The image reconstruction parameters were slice thickness 0.75 mm, increment 0.6 mm, and kernel I50f medium sharp. Evaluation of lung nodules was performed with computer-aided detection (CAD), using the software program MM oncology (Syngo.via, Siemens Healthineers, Erlangen, Germany). Nodule segmentation was performed using the commercially available software (Syngo.via version VB10B_HF03, Siemens Healthineers, Erlangen, Germany), providing estimates of nodule diameters and volume.^27^^–^^29^ For the present study, additional images with coronal MPR (slice thickness 3 mm, increment 2 mm) and coronal as well as transaxial MIPs (slice thickness 10 mm, slice spacing 0.6 mm) were reconstructed.

All DTS examinations were performed with a GE Definium 8000 system with a VolumeRAD option (GE Healthcare). Sixty low-dose projection images were collected in a linear sweep of the X-ray tube. A sweep angle of 30 deg and a stationary detector were used. Acquisition time was ∼11  s using a tube voltage of 120 kV and additional filtration of 3 mm Al + 0.1 mm Cu. The tube load (mAs) used for the collection of each projection image was determined from a posterior-anterior scout image collected using automatic exposure control. The DTS system multiplies the exposure obtained for the scout image with a factor of 10 and evenly distributes the resulting exposure over the 60 projection images included in the DTS examination. The exposure obtained for each projection image was rounded down to the closest mAs setting possible on the system. The coronal DTS section images were reconstructed with a 5-mm interval.

Twenty-four participants with solid lung nodules examined with CT and DTS on the same day were randomly selected for the present study. The CAD volume of the largest nodule in each participant ranged from 104 to $630\;{\mathrm{mm}}^{3}$ (median volume $144\;{\mathrm{mm}}^{3}$).

### 2.2 Study Design and Statistical Analysis

**Fig. 1:**
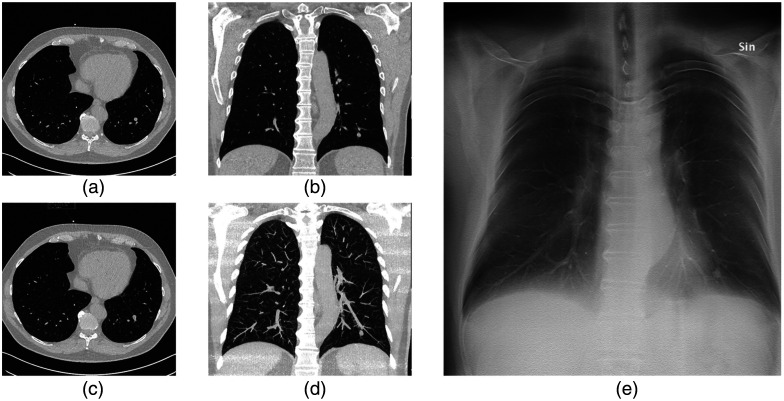
Illustration of the image display used in the study. The observers were instructed to select the largest nodule in the CAD series (c) and to mark and localize the nodule in all CT series and DTS (e) images using the transaxial (a), the coronal MPR (b), and the CT-MIP (d) series as reference. In the first review session, a coronal or transaxial MIP series were shown to the observers in a unique randomized order. Abbreviations; CT = computed tomography, CAD = computer-aided detection, MIP = maximum intensity projection, and DTS = digital tomosynthesis.

The present study entails three parts. In the first part, the preferred orientation of CT-MIP when localizing a nodule in DTS was evaluated. The second part assessed nodule localization and visibility in DTS compared with CT, and the third part concerned nodule size estimated by diameter and area measurements. Five radiologists with different levels of experience participated as observers in the study. Observers 1 and 2 were senior cardiothoracic consultant radiologists each with over 25 years of experience. Observer 3 was a senior cardiothoracic consultant radiologist with over 10 years of experience. Observers 4 and 5 were radiologists each with ∼5 years of experience. A medical doctor with a few months of training in radiology (observer 6) participated in the study, but the measurements of this observer were not compared with the other observers. The image viewer ViewDEX^30^^–^^33^ was used for image visualization and data collection, and all cases were evaluated in a randomized order, unique for each observer. An illustration of the image display used in the study is presented in Fig. 1. The study consisted of two review sessions, described in detail below. For each review session, the observers reviewed the cases using two monitors. The first monitor was divided into four canvases displaying transverse CT images, coronal CT images, CAD results (showing the location and size of the largest nodule in the case), and CT-MIP images (transaxial or coronal in review session 1 and only coronal in review session 2, for details, see below). The second monitor displayed the corresponding DTS.

### 2.3 Review Session 1

Three observers participated in the first review session (observers 1, 4, and 6) and reviewed all 24 cases twice: once with access to coronal CT-MIP and once with access to transaxial CT-MIP. Both the order of the cases and which orientation of the CT-MIP that were presented first were randomized for each observer. The task for the observers in review session 1 was to identify the largest nodule (shown by CAD on CT) and to localize and mark the nodule on CT images as well as on DTS images. The observers were also asked to grade to what extent the CT-MIP facilitated the localization of the largest nodule in the DTS images, using a five-point Likert scale (Table 1, Question 1). After identifying the nodule in the DTS images, the observers were instructed to grade the visibility of the nodule in DTS compared with coronal MPR CT images using a five-point Likert scale (Table 1, Questions 2 and 3) and to measure the area and diameter of the nodule in the DTS images. The latter was accomplished using a freehand region of interest (ROI) tool to draw the boundary area of the nodule and an electronic caliper to draw the longest and its perpendicular diameter of the nodule. The two diameter measurements were used to establish a nodule mD, in accordance with Fleischner Society Guidelines.^4^^,^^5^ After completion of the nodule measurements in DTS, the corresponding nodule diameter and area were measured in coronal CT.

**Table 1:** Questions to be answered by the observers. The first question (Q1) was used in the first part of the study, evaluating to what extent coronal and transaxial maximum intensity projections (MIPs) from computed tomography (CT) facilitated the localization of a lung nodule in digital tomosynthesis (DTS). The second and third questions (Q2 and Q3) were used in the second part of the study, evaluating localization and visibility of nodules in DTS and CT.

Question 1 (Q1)	MIP facilitated the localization of the nodule.
	1 = Confident that the statement is true.
	2 = Somewhat confident that the statement is true.
	3 = I do not know if the statement is true or not.
	4 = Somewhat confident that the statement is not true
	5 = Confident that the statement is not true.
Question 2 (Q2)	Confident that marking in DTS corresponds to CT.
	1 = Confident that the statement is true.
	2 = Somewhat confident that the statement is true.
	3 = I do not know if the statement is true or not.
	4 = Somewhat confident that the statement is not true.
	5= Confident that the statement is not true.
Question 3 (Q3)	The visibility of the nodule in MPR coronal (COR) and DTS
	1 = Definitely better in MPR COR.
	2 = Somewhat better in MPR COR.
	3 = Equally good in MPR COR and DTS.
	4 = Somewhat better in DTS.
	5 = Definitely better in DTS.

### 2.4 Review Session 2

All five radiologist observers (observers 1–5) participated in review session 2. In this session, the tasks of the observers were the same as in review session 1, except for the task of grading the extent to which the CT-MIP facilitated the localization of the nodule in DTS. The orientation of the CT-MIP shown to the observers during review session 2 was decided based on the results from review session 1. To reduce recall bias, the review of each session was separated in time by a minimum of 2 weeks.

### 2.5 Data Analysis

Prior to the data analysis, the pixel location on DTS and coordinates (x,y,z) on CT were checked to ensure that the same nodules were identified and measured by the observers.

The collected observer data from the two review sessions were analyzed using visual grading characteristics (VGC) analysis,^34^^,^^35^ Bland-Altman analysis,^36^ Wilcoxon signed-rank test, and descriptive statistics in three parts (see below).

#### 2.5.1 Part 1, coronal MIP versus transaxial MIP

A VGC analysis was performed for evaluation of the preferred orientation of CT-MIP in the task of localizing nodules in DTS based on the data acquired in review session 1. The statistical analysis was performed using VGC Analyzer,^37^^–^^39^ which determined the area under the VGC curve (AUC_VGC_) and its associated 95% confidence interval (CI). In the analysis, the coronal CT-MIP was used as the reference condition, meaning that an ${\mathrm{AUC}}_{\mathrm{VGC}}$ significantly below 0.5 (95% CI not covering 0.5) indicates that the coronal CT-MIP was rated as superior to transaxial CT-MIP and an ${\mathrm{AUC}}_{\mathrm{VGC}}$ significantly above 0.5 (95% CI not covering 0.5) indicates that the coronal CT-MIP was rated as inferior to transaxial CT-MIP. The analysis was performed based on a random reader assumption and the trapezoidal rule of curve fitting.

#### 2.5.2 Part 2, nodule localization and visibility of nodules

The confidence of nodule localization and visibility was assessed by descriptive statistics and presented in pie charts. The markings of the nodule in DTS and CT were confirmed by analyzing and comparing the marking coordinates in ViewDEX.

#### 2.5.3 Part 3, nodule size measurements

Initially, to assess if there were any significant differences between measuring mD and area on DTS and CT, respectively, a t-test assuming unequal variances was performed using Microsoft Excel©.

To be able to compare measurement variability between nodule mD and area measurements, a calculation of the volumes of the nodules was performed. In the case of diameter measurements, the mD of each nodule was used to calculate the volume of a sphere with the corresponding diameter, and in the case of area measurements, the volume of a sphere with a central section area corresponding to the measured nodule area was determined. Inter- and intra-observer agreements for nodule size estimates were evaluated according to Bland-Altman,^36^ presenting mean difference, the 95% CI, and limits of agreement (LoA). When assessing the inter-observer agreement for volumes based on mD and area, the results were presented considering the pair of observers (observers 1–5) with the best and worst agreement. Three observers (observers 1, 4, and 5) measured all nodules twice in review sessions 1 and 2. Intra-observer agreement for volumes based on mD and area was presented considering the observers with the best and worst agreement between their two rounds of measurements. To assess if there was a statistically significant difference in inter-observer variability between the mD-based volumes and area-based volumes, the standard deviation (SD) of the nodule measurements of the five observers (observers 1–5) was calculated for each nodule, both for the mD-based volumes and for the area-based volumes. For statistical analysis of the difference, a Wilcoxon signed-rank test was applied using Ref. 40. Additional linear regression calculations including coefficient of determination ($R^{2}$) and standard error of the estimate (SEE) were performed using Microsoft Excel©.

Finally, the nodule volumes from CAD were compared with the nodule volumes based on measurements of diameter and area on coronal CT from observers 1–5.

### 2.6 Radiation Dose Calculations

After a DTS examination, the raw-data projection images containing detailed exposure data are usually not stored in the picture archiving and communication system (PACS). Båth et al.^41^ have therefore presented a method that can be used to retrospectively estimate radiation doses resulting from DTS examinations using the VolumeRAD system (GE Healthcare, Chalfont St Giles, United Kingdom). The method was used in the present study and is based on using detailed exposure information from the scout image to estimate the total dose-area product (DAP) of the entire chest DTS examination. After determining the total DAP for each DTS examination included in the study using the method by Båth et al.,^41^ a conversion factor between DAP and effective dose of $0.26\;\mathrm{mSv}\;{\mathrm{Gy}}^{-1}\;{\mathrm{cm}}^{-2}$ ^42^ was applied to estimate the resulting effective dose from the examinations.

To estimate the effective dose resulting from the CT examinations included in the study, the dose-length product (DLP) value for each CT examination was multiplied with the conversion factor 0.015  mSv/mGy·cm.^43^

## 3 Results

### 3.1 Part 1, Coronal MIP Versus Transaxial MIP

The visual grading analysis showed that coronal CT-MIP was the preferred orientation for assistance of nodule localization in DTS (${\mathrm{AUC}}_{\mathrm{VGC}}=0.1$, asymmetric CI 0.0–0.1).

### 3.2 Part 2, Nodule Localization and Visibility of Nodules

**Fig. 2:**
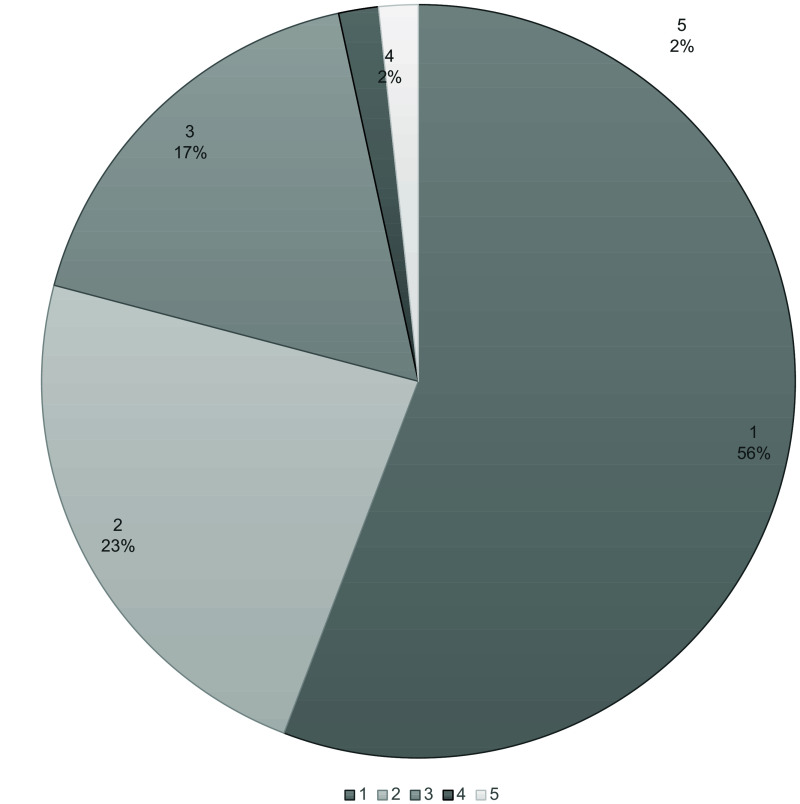
Results of the question “confident that marking in DTS corresponds to the marking CT.” 1 = Confident that the statement is true. 2 = Somewhat confident that the statement is true. 3 = I do not know if the statement is true or not. 4 = Somewhat confident that the statement is not true. 5 = Confident that the statement is not true. Abbreviations; DTS = digital tomosynthesis and CT = computed tomography.

In most cases (79%), the observers were confident or somewhat confident that their marked nodule on DTS corresponded to their nodule marked on CT, as shown in Fig. 2. The markings were confirmed by coordinates in ViewDEX, and only one nodule was wrongly marked on DTS by one of the observers. One observer could not identify the nodule on DTS in two cases. Hence, only 21 of the 24 nodules were included in the further analysis.

**Fig. 3:**
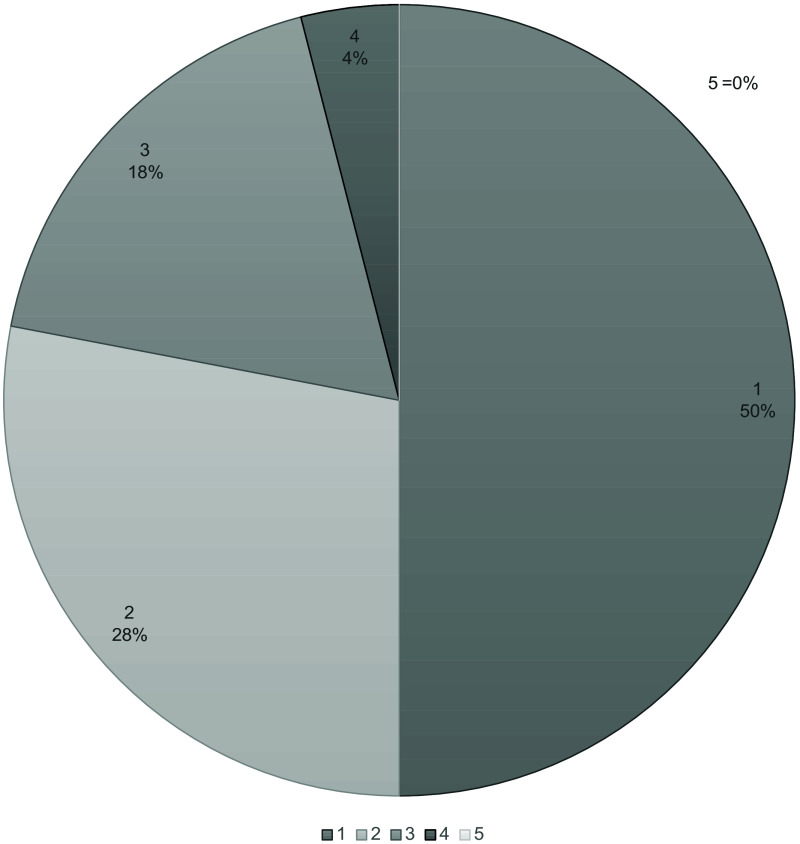
Results of the question “The visibility of the nodule in MPR COR and DTS.” 1 = Definitely better in MPR COR. 2 = Somewhat better in MPR COR. 3 = Equally good in MPR COR and DTS. 4 = Somewhat better in DTS. 5 = Definitely better in DTS. Abbreviations; MPR = multiplanar reformation, COR= coronal, and DTS = digital tomosynthesis.

The vast majority of the observers considered that the nodules were better visualized on coronal MPR CT images than in DTS (Fig. 3). However, one of the experienced observers considered five nodules to be somewhat better visualized on DTS in comparison to CT.

### 3.3 Part 3, Nodule Measurement

**Fig. 4:**
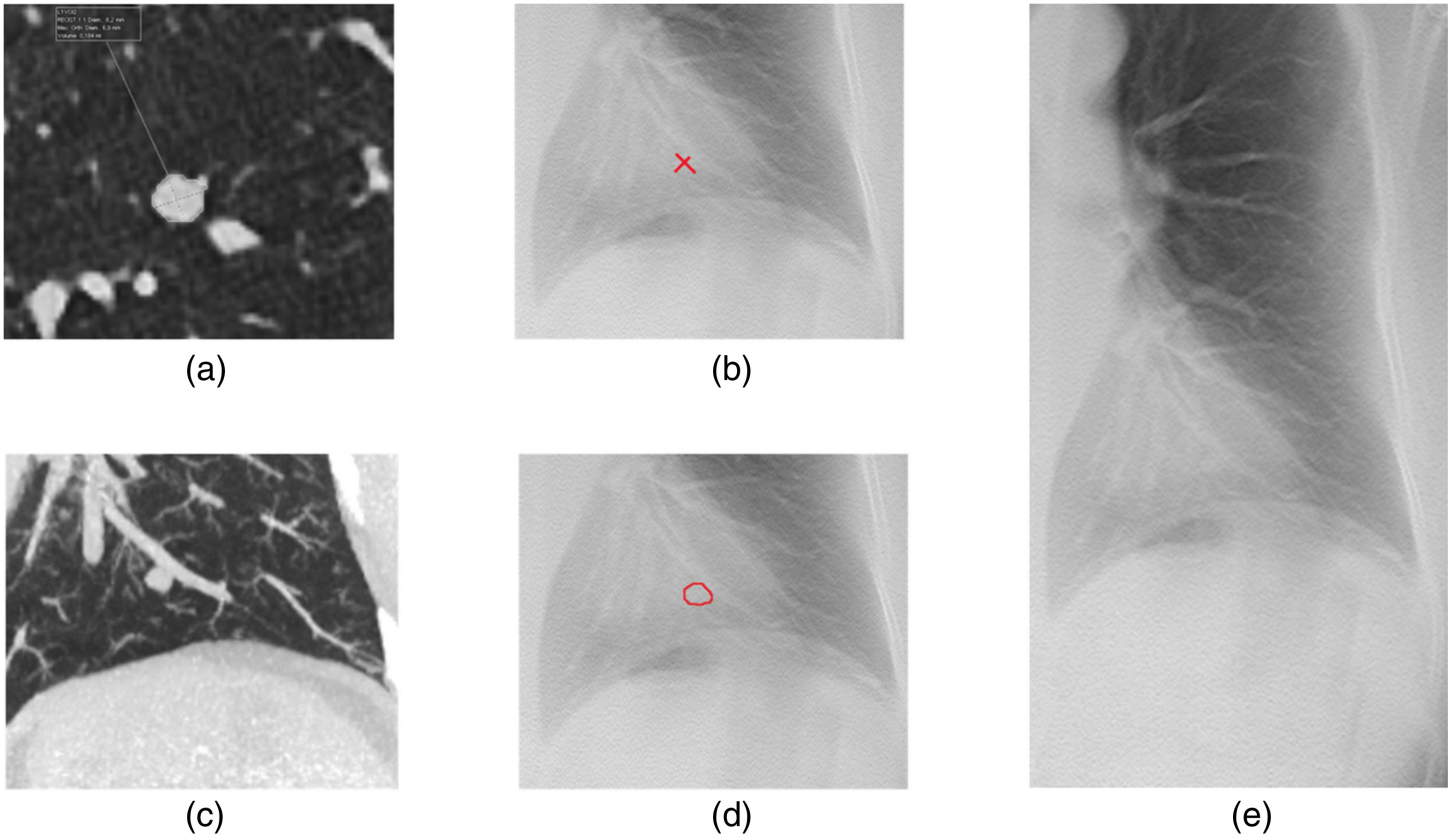
Illustration of the same nodule on CT and DTS (one case out of 24). (a) The selected nodule on a CAD axial view. (b) The selected nodule on DTS (2 diameter measurements used to calculate the mD in red). (c) The selected nodule on a coronal MIP. (d) The selected nodule on DTS (drawn area in red). (e) The selected nodule on DTS. Abbreviations; CT = computed tomography, DTS = digital tomosynthesis, CAD = computer-aided detection, mD = mean diameter, and MIP = maximum intensity projection.

Three nodules out of 24 were not considered measurable by all the observers. Consequently, diameter and area measurements of 21 nodules performed by five observers were included in the analysis. An illustration of the measurement of a representative nodule is given in Fig. 4.

Concerning linear measurement, there were no statistically significant differences between measuring the mD (p=0.40) and the area (p=0.68) on DTS or CT, respectively.

**Fig. 5:**
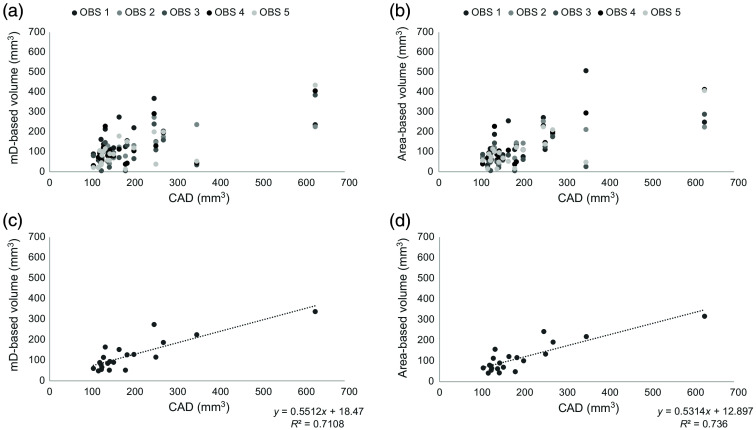
Scatter plots of the lung nodule volume estimate on DTS of the chest based on mD-based nodule volume (a) and area-based nodule volume (b) of the five observers in comparison to CAD-derived nodule volumes. Scatter plots of the lung nodule volume estimate on DTS of the chest based on mD-based nodule volume (average of the five observers) with a SEE 42,7 (c), and area-based nodule volume (average of the five observers) with a SEE 38,7 (d) of the five observers in comparison to CAD-derived nodule volumes and respective linear regressions. Abbreviations; DTS = digital tomosynthesis, mD = mean diameter, CAD = computer-aided detection, SEE = standard error of the estimate, and $R^{2}$ = coefficient of determination.

Scatter plots showing the nodule volumes based on mD measurements and area measurements compared with the nodule volumes obtained by CT-CAD are presented in Fig. 5.

**Fig. 6:**
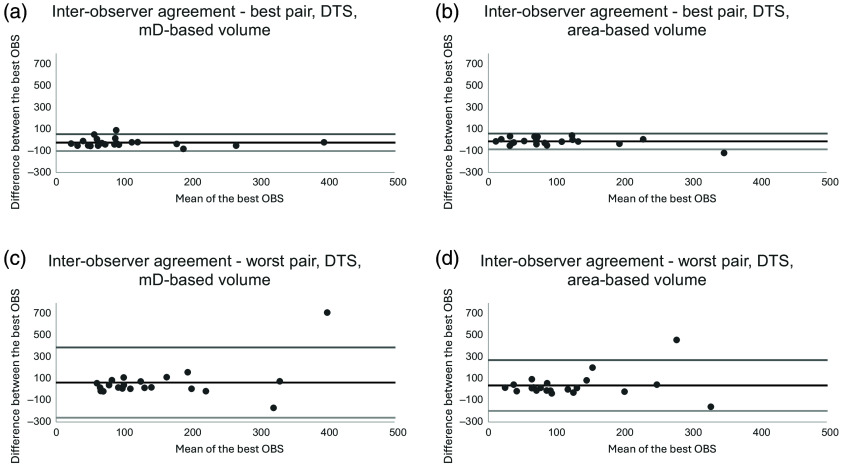
Bland-Altman plots of the best (a) and (b) and worst (c) and (d) inter-observer agreement when assessing measurements of mD-based nodule volume and area-based nodule volume on DTS of the chest. Abbreviations: mD = mean diameter and DTS = digital tomosynthesis.

When assessing the inter-observer agreement for volumes based on mD measurements, the mean difference ranged from −23.7 (CI−40.2 to −7.2; LoA−101.0 to 53.6) ${\mathrm{mm}}^{3}$ for the best inter-observer agreement to 65.1 (CI−4.4 to 134.6; LoA−259.4 to 389.7) ${\mathrm{mm}}^{3}$ for the worst inter-observer agreement. When assessing the corresponding inter-observer agreement for volumes based on area measurements, the mean difference ranged from −12.7 (CI−28.2 to 2.8; LoA −85.2 to 59.8) ${\mathrm{mm}}^{3}$ for the best inter-observer agreement to 38.1 (CI−12.1 to 88.2; LoA −196.4 to 272.5) ${\mathrm{mm}}^{3}$ for the worst inter-observer agreement. Bland-Altman plots showing the agreement are presented in Fig. 6.

The performed Wilcoxon signed-rank test showed that the measurement variability was significantly larger when calculating nodule volume based on mD measurements in comparison to nodule volume based on area measurements (p<0.05).

**Fig. 7:**
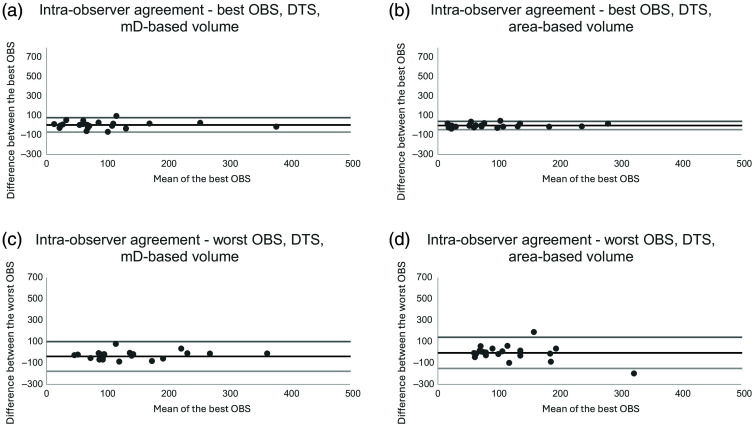
Bland-Altman plots of the best (a) and (b) and worst (c) and (d) intra-observer agreement when assessing measurements of mD-based nodule volume and area-based nodule volume on DTS of the chest. Abbreviations: mD = mean diameter and DTS = digital tomosynthesis.

Three observers (observers 1, 4, and 5) measured all nodules twice (review session 1 and review session 2). These data were used to assess the intra-observer agreement of the measurements. For nodule volumes based on mD measurements, the mean difference in intra-observer agreement ranged from 4.6 (CI−11.2 to 20.4; LoA−69.5 to 78.7) ${\mathrm{mm}}^{3}$ for the best intra-observer agreement to −38.0 (CI−67.7 to −8.2; LoA−177.1 to 101.2) ${\mathrm{mm}}^{3}$ for the worst intra-observer agreement. When assessing the corresponding intra-observer agreement for nodule volumes based on area measurements, the mean difference ranged from −1.3 (CI−10.4 to 7.7; LoA−43.8 to 41.1) ${\mathrm{mm}}^{3}$ for the best intra-observer agreement to −4.0 (CI−35.2 to 27.2; LoA −150.0 to $142.0\;{\mathrm{mm}}^{3}$ for the worst intra-observer agreement. Bland-Altman plots showing the agreement are presented in Fig. 7.

### 3.4 Agreement of Volume Estimates on DTS in Comparison with CAD Volumes from CT

Regarding nodule volume based on mD measurements on DTS, the agreement with CT-CAD volumes ranged from a mean difference of −85.7 (CI−117.7 to −53.8; LoA−235.1 to 63.7) ${\mathrm{mm}}^{3}$ for the best agreement to −9.1 (CI−68.8 to 50.6; LoA−288.3 to 270.0) ${\mathrm{mm}}^{3}$ for the worst agreement.

Regarding nodule volume based on area measurement on DTS, the agreement with CT-CAD volumes ranged from a mean difference of −64.2 (CI −89.8 to −38.6; LoA−183.9 to 55.5) ${\mathrm{mm}}^{3}$ for the best agreement to −51.7 (CI−95.9 to 7.6; LoA−258.3 to 154,8) ${\mathrm{mm}}^{3}$ for the worst agreement.

The study design also included an observer with minor experience in radiology, this medical doctor (observer 6) presented, in comparison to CAD, a mean difference of −105.0 (CI−143.8 to −66.7; LoA−285.6 to 75.1) ${\mathrm{mm}}^{3}$ regarding nodule volume based on mD, and a mean difference of −104.0 (CI−13.19 to −68.6; LoA−268.8 to 61.0) ${\mathrm{mm}}^{3}$ regarding nodule volume based on area measurement.

### 3.5 Agreement of Volume Estimates on CT in Comparison with CAD Volumes from CT

Regarding nodule volume based on mD measurements on CT (coronal plane), the agreement with CT-CAD volumes ranged from a mean difference of −81.3 (CI−109.7 to −53.0; LoA−214.1 to 51.4) ${\mathrm{mm}}^{3}$ for the best agreement to −58.9 (CI−98.3 to −19.4; LoA−243.3 to 125.6) ${\mathrm{mm}}^{3}$ for the worst agreement.

Regarding nodule volume based on area measurement on CT (coronal plane), the agreement with CT-CAD volumes ranged from a mean difference of −60.0 (CI−89.0 to −31.0; LoA −195.6 to 75.6) ${\mathrm{mm}}^{3}$ for the best agreement to −82.6 (CI−116.6 to −48.6; LoA−241.5 to 76.2) ${\mathrm{mm}}^{3}$ for the worst agreement.

The medical doctor with limited training in radiology (observer 6) presented, in comparison to CAD, a mean difference of −101.3 (CI−137.9 to −64.8; LoA−272.3 to 69.7) ${\mathrm{mm}}^{3}$ regarding nodule volume based on mD, and a mean difference of −96.7 (CI−137.4 to −56.0; LoA−286.8 to 93.4) ${\mathrm{mm}}^{3}$ regarding nodule volume based on area measurement.

### 3.6 Radiation Dose

The average effective dose per DTS in the present study was 0.2 mSv (minimum of 0.1 mSv, maximum of 0.3 mSv). The average effective dose per CT in the present study was 0.7 mSv (minimum of 0.5 mSv, maximum of 1.0 mSv). Scout images were not included in the dose estimates for DTS or CT.

## 4 Discussion

The findings in the present study indicate that coronal CT-MIPs are the preferred orientation when localizing lung nodules known from CT in DTS images, that most nodules detected with CT are visible in DTS, and that measurements of nodule area on DTS result in less measurement variability than measurements of nodule diameter.

In previous studies, CT-MIP have been shown to be a useful tool to help radiologists detect lung nodules on CT.^44^^–^^47^ However, further research on the preferred orientation of the CT-MIP was needed to specifically address the usefulness of CT-MIP in the process of identifying nodules on DTS. In this study, coronal CT-MIP images were considered to facilitate the localization of the nodule in DTS and were rated by the observers as superior to transaxial CT-MIP images, at a strong statistical significance level.

Concerning the localization and visibility of lung nodules on DTS, the observers were highly confident in their ability to localize the same nodules on DTS and on CT (see Fig. 2), even though the visibility of the nodules was rated higher in the CT images than in the DTS images (see Fig. 3). Most of the lung nodules could be identified on DTS regardless of observer experience. These results are in line with the results from other studies. For example, in a study by Lee et al.,^48^ focusing on lung nodule visibility on DTS and learning curves associated with DTS reading times, it was shown that most malignant nodules >5  mm were visible on DTS.

The fact that five nodules were considered somewhat better visualized on DTS when compared with coronal CT could possibly be explained by their more central location in the coronal plane on DTS. It is known that DTS presents better spatial resolution in the x−y plane (coronal plane) than CT.^49^ This benefit may however be obscured by blurring from out-of-plane objects caused by the limited depth resolution in reconstructed DTS images. According to Machida et al.,^50^ the blurring effect increases if the structures are denser and located perpendicular to the sweep angle and decreases with increasing distance from the dense object. Consequently, the visualization of nodules with a more central location in the reconstructed volume may be less affected by disturbing blurring from, e.g., ribs compared with nodules located close to the rib cage and therefore benefit more from the higher in-plane resolution in DTS compared with CT.

It has previously been suggested that DTS might have the potential to be a modality that could be used in lung cancer screening^51^ as well as for lung nodule follow-up.^9^ The benefits of DTS in terms of lower radiation and lower cost when compared with CT could promote the use of DTS as an alternative screening method.^52^ The recent European policy statement encourages countries to implement lung cancer screening,^53^ and low-dose CT is the primary diagnostic method for lung cancer screening.^54^ However, Bertolaccini et al. suggested that DTS could be used as the first-line lung cancer screening modality,^52^ which could be an alternative if CT resources are scarce.

Determination of nodule size is crucial in lung cancer screening. Previous studies have shown that measurement variability is a concern in DTS.^9^ Nodule volume is the preferred size estimate in lung cancer screening as well as for lung nodule follow-up.^16^ Unfortunately, volume cannot be measured on DTS. Measurements of diameter and or area should therefore be considered. In a study by Söderman et al.,^55^ it was shown that there is a risk for underestimation of lung nodule size when lung nodule diameter is manually measured, especially if in-plane artifacts are present in the images. In-plane artifacts are perceived as a darker rim around for example a lung nodule, related to the direction of the scan. As the artifact is more prominent in the scan direction, it can be anticipated that the effect of the artifact on nodule size determination is larger for diameter measurements than for area measurements. Consequently, area-based measurements may be a way to decrease the measurement variability. This hypothesis is supported by the results of the current study. It should be noted that in the case of nodule follow-up, it is recommended by RECIST guidelines^56^ that the same observer measures the nodule using the same modality and measurement technique. This is a well-known method to avoid inter-modality and inter-observer variability.

Even though measurements of nodule size in CT and DTS cannot be used interchangeably,^57^ it might still be possible to use DTS for follow-up of nodules detected on DTS in a screening scenario. It has been shown that manual measurements on DTS have comparable repeatability when compared with manual measurements on CT and that no bias existed between the two modalities.^57^ Most nodules in the present study were rated as visible in DTS in contrast to the study by Lee et al.^48^ where only 53.3% of the nodules were reported as visible on DTS. The results in the present study probably reflect the inclusion of larger nodules in comparison to the previously mentioned study^48^ and the access to CT images used as a reference for nodule location. Even if all the observers considered that the nodules were better visualized on coronal MPR CT images compared with DTS, some nodules were at least equally visible on DTS. Regarding the three nodules that were not measured by all the observers, one observer measured another nodule positioned close to the nodule measured by the other four observers and one out of five observers considered two nodules non-measurable, which might be due to lesser contrast in the DTS images in comparison to CT. However, the reason is at the discretion of this observer.

It should also be noted that factors such as different patient positionings, nodule sizes, and dose levels during different scan occasions (baseline and follow-ups) could interfere with the precision of the measurements performed on DTS, as described by Söderman et al.^58^

The present study aims at investigating nodule size estimates in a situation that resembles clinical praxis. Due to the limited depth resolution in DTS, there is little relevance in reconstructing section images using an interval smaller than 5 mm, whereas CT images frequently are reconstructed with a slice thickness of 3 mm with a 2-mm interval. Nevertheless, this limited depth resolution on DTS will affect the size measurements.

The present study had the strength of having a decent number of observers, and the examinations were carried out on the same day and on the same equipment. Furthermore, the present study benefited from having observers with different experiences in the field of radiology, with a range of experience extending from just some months of training in radiology to many years of experience in thoracic radiology. Our results indicate that even an observer with minor experience in the field of radiology can perform measurements on DTS comparable to radiologists. In addition, this study benefited from the fact the images were visualized with anonymization of the cases and in a unique randomized order for each observer. However, there were also several limitations in the present study. The numbers regarding both cases and nodules were low, and consequently, the number of nodules in each size category was limited. Only solid lung nodules were included, and the measuring tool used in the present study was new to the observers. Also, even if our study shows that most intermediate or large-size nodules (nodules greater than or equal to $100\;{\mathrm{mm}}^{3}$) that were found on CT could be visualized, accurately localized, and correctly measured in DTS, the assumption made in the present study that lung nodules are sphere-like structures will introduce an uncertainty in the resulting estimated nodule volumes. Using this assumption, even small differences in measured diameters and areas might result in considerable differences in resulting nodule volume. For example, if one observer measures the diameter of a specific nodule in DTS to be 6 mm and a different observer measures the same nodule diameter to be 7 mm, the assumption that the lung nodules are sphere-like structures results in estimated nodule volumes of 113 and $179.6\;{\mathrm{mm}}^{3}$ for the 6-mm-diameter nodule and the 7-mm-diameter nodule, respectively. Hence, there will be a large difference in resulting nodule volume despite only a 1-mm difference in measured nodule diameter. Using area-based measurements instead of diameter-based measurements for the estimation of the nodule volumes in DTS improves the agreement of the estimated nodule volumes between DTS and CT, but even with area-based volume estimations on DTS, the method might still introduce considerable differences in nodule volumes between DTS and CT.

To summarize, assessing lung nodules with DTS would be a way to reduce patient exposure to radiation and the costs associated with lung nodule assessment. Estimating lung nodule size through area measurements at screening and follow-up could be a key to reduce inter-observer variation on DTS. The results of the present study will be further explored in a larger cohort of participants with lung nodules in SCAPIS.

## 5 Conclusions

The present study shows that coronal CT-MIP images are superior to transaxial CT-MIP images in facilitating lung nodule localization in DTS. Furthermore, most nodules with a size larger than $100\;{\mathrm{mm}}^{3}$ found on CT can be visualized, correctly localized, and measured in DTS. Finally, when assessing nodule size in DTS, area measurements might result in less variability in comparison to diameter measurements.

## Data Availability

All data in support of the findings of this paper are available within the article or may be requested as supplementary material.

## References

[1] Dobbins J. T. 3rd et al., “Digital tomosynthesis of the chest,” J. Thorac. Imaging 23(2), 86–92 (2008). JTIME8 0883-5993 10.1097/RTI.0b013e318173e162 18520565

[2] Ferrari A. et al., “Digital chest tomosynthesis: the 2017 updated review of an emerging application,” Ann. Transl. Med. 6(5), 91 (2018). 10.21037/atm.2017.08.18 29666814 PMC5890049

[3] Hansell D. M. et al., “Fleischner Society: glossary of terms for thoracic imaging,” Radiology 246(3), 697–722 (2008). RADLAX 0033-8419 10.1148/radiol.2462070712 18195376

[4] Bueno J. Landeras L. Chung J. H., “Updated Fleischner Society guidelines for managing incidental pulmonary nodules: common questions and challenging scenarios,” Radiographics 38(5), 1337–1350 (2018). 10.1148/rg.2018180017 30207935

[5] MacMahon H. et al., “Guidelines for management of incidental pulmonary nodules detected on CT images: from the Fleischner Society 2017,” Radiology 284(1), 228–243 (2017). RADLAX 0033-8419 10.1148/radiol.2017161659 28240562

[r6] Callister M. E. et al., “British Thoracic Society guidelines for the investigation and management of pulmonary nodules,” Thorax 70(Suppl 2), ii1–ii54 (2015). THORA7 0040-6376 10.1136/thoraxjnl-2015-207168 26082159

[7] Gould M. K. et al., “Evaluation of individuals with pulmonary nodules: when is it lung cancer? Diagnosis and management of lung cancer, 3rd ed: American College of Chest Physicians evidence-based clinical practice guidelines,” Chest 143(5 Suppl), e93S–e120S (2013). CHETBF 0012-3692 10.1378/chest.12-2351 23649456 PMC3749714

[8] Cao C. F. et al., “CT scans and cancer risks: a systematic review and dose-response meta-analysis,” BMC Cancer 22(1), 1238 (2022). BCMACL 1471-2407 10.1186/s12885-022-10310-2 36451138 PMC9710150

[9] Meltzer C. et al., “Surveillance of small, solid pulmonary nodules at digital chest tomosynthesis: data from a cohort of the pilot Swedish CArdioPulmonary bioImage Study (SCAPIS),” Acta Radiol. 62(3), 348–359 (2021). 10.1177/0284185120923106 32438877 PMC7930602

[10] Langer S. G. et al., “Sensitivity of thoracic digital tomosynthesis (DTS) for the identification of lung nodules,” J. Digital Imaging 29(1), 141–147 (2016). 10.1007/s10278-015-9818-0 PMC4722026 26349914

[11] Machida H. et al., “Whole-body clinical applications of digital tomosynthesis,” Radiographics 36(3), 735–750 (2016). 10.1148/rg.2016150184 27163590

[12] Larici A. R. et al., “Lung nodules: size still matters,” Eur. Respir. Rev. 26(146), 170025 (2017). EREWEH 10.1183/16000617.0025-2017 29263171 PMC9488618

[13] Naidich D. P. et al., “Recommendations for the management of subsolid pulmonary nodules detected at CT: a statement from the Fleischner Society,” Radiology 266(1), 304–317 (2013). RADLAX 0033-8419 10.1148/radiol.12120628 23070270

[14] Revel M. P. et al., “Are two-dimensional CT measurements of small noncalcified pulmonary nodules reliable?,” Radiology 231(2), 453–458 (2004). RADLAX 0033-8419 10.1148/radiol.2312030167 15128990

[15] Yankelevitz D. F. et al., “Small pulmonary nodules: evaluation with repeat CT–preliminary experience,” Radiology 212(2), 561–566 (1999). RADLAX 0033-8419 10.1148/radiology.212.2.r99au33561 10429718

[16] Devaraj A. et al., “Use of volumetry for lung nodule management: theory and practice,” Radiology 284(3), 630–644 (2017). RADLAX 0033-8419 10.1148/radiol.2017151022 28825886

[17] Nair A. et al., “Contextualizing the role of volumetric analysis in pulmonary nodule assessment: AJR expert panel narrative review,” Am. J. Roentgenol. 220(3), 314–329 (2023). AJROAM 0092-5381 10.2214/AJR.22.27830 36129224

[18] Guedes Pinto E. et al., “Factors influencing the outcome of volumetry tools for pulmonary nodule analysis: a systematic review and attempted meta-analysis,” Insights Imaging 14(1), 152 (2023). 10.1186/s13244-023-01480-z 37741928 PMC10517915

[19] Bankier A. A. et al., “Recommendations for measuring pulmonary nodules at CT: a statement from the Fleischner Society,” Radiology 285(2), 584–600 (2017). RADLAX 0033-8419 10.1148/radiol.2017162894 28650738

[20] Meltzer C. et al., “Detection and characterization of solid pulmonary nodules at digital chest tomosynthesis: data from a cohort of the Pilot Swedish Cardiopulmonary Bioimage Study,” Radiology 287(3), 1018–1027 (2018). RADLAX 0033-8419 10.1148/radiol.2018171481 29613826

[r21] Dobbins J. T. 3rd et al., “Multi-institutional evaluation of digital tomosynthesis, dual-energy radiography, and conventional chest radiography for the detection and management of pulmonary nodules,” Radiology 282(1), 236–250 (2016). RADLAX 0033-8419 10.1148/radiol.2016150497 27439324 PMC5207128

[22] Kumar S. G. et al., “Role of digital tomosynthesis and dual energy subtraction digital radiography in detecting pulmonary nodules,” Eur. J. Radiol. 84(7), 1383–1391 (2015). EJRADR 0720-048X 10.1016/j.ejrad.2015.03.020 25892052

[23] Jabeen N. et al., “Diagnostic accuracy of maximum intensity projection in diagnosis of malignant pulmonary nodules,” Cureus 11(11), e6120 (2019). 10.7759/cureus.6120 31886058 PMC6903899

[24] Valencia R. et al., “Value of axial and coronal maximum intensity projection (MIP) images in the detection of pulmonary nodules by multislice spiral CT: comparison with axial 1-mm and 5-mm slices,” Eur. Radiol. 16(2), 325–332 (2006). 10.1007/s00330-005-2871-1 16086181

[25] Bergstrom G. et al., “The Swedish CArdioPulmonary BioImage Study: objectives and design,” J. Intern. Med. 278(6), 645–659 (2015). 10.1111/joim.12384 26096600 PMC4744991

[26] Toren K. et al., “Vital capacity and COPD: the Swedish CArdioPulmonary bioImage Study (SCAPIS),” Int. J. Chron. Obstruct. Pulmon. Dis. 11, 927–933 (2016). 10.2147/COPD.S104644 27194908 PMC4859418

[27] Heckel F. et al., “Partial volume correction for volume estimation of liver metastases and lymph nodes in CT scans using spatial subdivision,” Proc. SPIE 7623, 76230T (2010), PSISDG 0277-786X 10.1117/12.844200

[r28] Kuhnigk J. M. et al., “Morphological segmentation and partial volume analysis for volumetry of solid pulmonary lesions in thoracic CT scans,” IEEE Trans. Med. Imaging 25(4), 417–434 (2006). ITMID4 0278-0062 10.1109/TMI.2006.871547 16608058

[29] Moltz J. H. et al., “Advanced segmentation techniques for lung nodules, liver metastases, and enlarged lymph nodes in CT scans,” IEEE J. Sel. Top. Signal Process. 3(1), 122–134 (2009). 10.1109/JSTSP.2008.2011107

[30] Svalkvist A. et al., “Viewdex 3.0-recent development of a software application facilitating assessment of image quality and observer performance,” Radiat. Prot. Dosim. 195(3–4), 372–377 (2021). RPDODE 0144-8420 10.1093/rpd/ncab014 PMC8507463 33683321

[r31] Svalkvist A. et al., “Viewdex: a status report,” Radiat. Prot. Dosim. 169(1–4), 38–45 (2016). RPDODE 0144-8420 10.1093/rpd/ncv543 26822421

[r32] Hakansson M. et al., “VIEWDEX: an efficient and easy-to-use software for observer performance studies,” Radiat. Prot. Dosim. 139(1–3), 42–51 (2010). RPDODE 0144-8420 10.1093/rpd/ncq057 20200105

[33] Borjesson S. et al., “A software tool for increased efficiency in observer performance studies in radiology,” Radiat. Prot. Dosim. 114(1–3), 45–52 (2005). RPDODE 0144-8420 10.1093/rpd/nch550 15933080

[34] Bath M. Mansson L. G., “Visual grading characteristics (VGC) analysis: a non-parametric rank-invariant statistical method for image quality evaluation,” Br. J. Radiol. 80(951), 169–176 (2007). BJRAAP 0007-1285 10.1259/bjr/35012658 16854962

[35] Bath M., “Evaluating imaging systems: practical applications,” Radiat. Prot. Dosim. 139(1–3), 26–36 (2010). RPDODE 0144-8420 10.1093/rpd/ncq007 20147386

[36] Bland J. M. Altman D. G., “Statistical methods for assessing agreement between two methods of clinical measurement,” Lancet 1(8476), 307–310 (1986). 10.1016/S0140-6736(86)90837-8 2868172

[37] Bath M. Hansson J., “VGC analyzer: a software for statistical analysis of fully crossed multiple-reader multiple-case visual grading characteristics studies,” Radiat. Prot. Dosim. 169(1–4), 46–53 (2016). RPDODE 0144-8420 10.1016/S0140-6736(86)90837-8 26769908

[r38] Hansson J. Mansson L. G. Bath M., “The validity of using ROC software for analysing visual grading characteristics data: an investigation based on the novel software VGC analyzer,” Radiat. Prot. Dosim. 169(1–4), 54–59 (2016). RPDODE 0144-8420 10.1093/rpd/ncw035 26979808

[39] Hansson J. Mansson L. G. Bath M., “Evaluation of VGC analyzer by comparison with gold standard ROC software and analysis of simulated visual grading data,” Radiat. Prot. Dosim. 195(3–4), 378–390 (2021). 10.1093/rpd/ncab066 PMC8507457 33940628

[40] “The Wilcoxon Signed-Ranks Test Calculator,” Social Science Statistics, 2024, https://www.socscistatistics.com/tests/signedranks/default.aspx (accessed 2024).

[41] Bath M. Soderman C. Svalkvist A., “A simple method to retrospectively estimate patient dose-area product for chest tomosynthesis examinations performed using VolumeRAD,” Med. Phys. 41(10), 101905 (2014). MPHYA6 0094-2405 10.1118/1.4895002 25281957

[42] Bath M. et al., “Effective dose to patients from chest examinations with tomosynthesis,” Radiat. Prot. Dosim. 139(1–3), 153–158 (2010). RPDODE 0144-8420 10.1093/rpd/ncq092 20233755

[43] Deak P. D. Smal Y. Kalender W. A., “Multisection CT protocols: sex- and age-specific conversion factors used to determine effective dose from dose-length product,” Radiology 257(1), 158–166 (2010). RADLAX 0033-8419 10.1148/radiol.10100047 20851940

[44] Kawel N. et al., “Effect of slab thickness on the CT detection of pulmonary nodules: use of sliding thin-slab maximum intensity projection and volume rendering,” Am. J. Roentgenol. 192(5), 1324–1329 (2009). AJROAM 0092-5381 10.2214/AJR.08.1689 19380557

[r45] Park E. A. et al., “Efficacy of computer-aided detection system and thin-slab maximum intensity projection technique in the detection of pulmonary nodules in patients with resected metastases,” Invest. Radiol. 44(2), 105–113 (2009). INVRAV 0020-9996 10.1097/RLI.0b013e318190fcfc 19034026

[r46] Kilburn-Toppin F. et al., “Detection of pulmonary nodules at paediatric CT: maximum intensity projections and axial source images are complementary,” Pediatr. Radiol. 43(7), 820–826 (2013). PDRYA5 1432-1998 10.1007/s00247-012-2597-6 23344916

[47] Li W. J. et al., “Effect of slab thickness on the detection of pulmonary nodules by use of CT maximum and minimum intensity projection,” Am. J. Roentgenol. 213(3), 562–567 (2019). AJROAM 0092-5381 10.2214/AJR.19.21325 31063429

[48] Lee K. H. et al., “Digital tomosynthesis for evaluating metastatic lung nodules: nodule visibility, learning curves, and reading times,” Korean J. Radiol. 16(2), 430–439 (2015). 10.3348/kjr.2015.16.2.430 25741205 PMC4347279

[49] Dobbins J. T. 3rd McAdams H. P., “Chest tomosynthesis: technical principles and clinical update,” Eur. J. Radiol. 72(2), 244–251 (2009). EJRADR 0720-048X 10.1016/j.ejrad.2009.05.054 19616909 PMC3693857

[50] Machida H. et al., “Optimizing parameters for flat-panel detector digital tomosynthesis,” Radiographics 30(2), 549–562 (2010). 10.1148/rg.302095097 20228334

[51] Terzi A. et al., “Lung cancer detection with digital chest tomosynthesis: baseline results from the observational study SOS,” J. Thorac. Oncol. 8(6), 685–692 (2013). 10.1097/JTO.0b013e318292bdef 23612466

[52] Bertolaccini L. et al., “Lung cancer detection with digital chest tomosynthesis: first round results from the SOS observational study,” Ann. Transl. Med. 3(5), 1–6 (2015). 10.3978/j.issn.2305-5839.2015.03.41 25992366 PMC4402610

[53] Council of the European Union, “Council Recommendation of 9 December 2022 on strengthening prevention through early detection: a new EU approach on cancer screening replacing Council Recommendation 2003/878/EC,” Off. J. Eur. Union 473, 1–10 (2022).

[54] Wood D. E. et al., “Lung cancer screening, version 3.2018, NCCN Clinical practice guidelines in oncology,” J. Natl. Compr. Cancer Network 16(4), 412–441 (2018). 10.6004/jnccn.2018.0020 PMC6476336 29632061

[55] Soderman C. et al., “Influence of the in-plane artefact in chest tomosynthesis on pulmonary nodule size measurements,” Radiat. Prot. Dosim. 169(1–4), 199–203 (2016). RPDODE 0144-8420 10.1093/rpd/ncv536 26769904

[56] Eisenhauer E. A. et al., “New response evaluation criteria in solid tumours: revised RECIST guideline (version 1.1),” Eur. J. Cancer 45(2), 228–247. (2009). EJCAEL 0959-8049 10.1016/j.ejca.2008.10.026 19097774

[57] Johnsson Å. A. et al., “Pulmonary nodule size evaluation with chest tomosynthesis,” Radiology 265(1), 273–282 (2012). RADLAX 0033-8419 10.1148/radiol.12111459 22993220

[58] Soderman C. et al., “Detection of pulmonary nodule growth with chest tomosynthesis: a human observer study using simulated nodules,” Acad. Radiol. 26(4), 508–518 (2019). 10.1016/j.acra.2018.05.004 29903641

